# Gender Differences in Depressive Symptoms and Work Environment Factors among Dairy Farmers in Japan

**DOI:** 10.3390/ijerph17072569

**Published:** 2020-04-09

**Authors:** Miho Sato, Hiromi Kato, Makiko Noguchi, Hiroshi Ono, Kuniyuki Kobayashi

**Affiliations:** 1 Faculty of Health Sciences, Hokkaido University, Sapporo 060-0812, Japan; 2 Research Faculty of Agriculture, Hokkaido University, Sapporo 060-8589, Japan; hkato@cen.agr.hokudai.ac.jp (H.K.); kobakuni@cen.agr.hokudai.ac.jp (K.K.); 3 Japanese Red Cross College of Nursing, Tokyo 150-0012, Japan; m-noguchi@redcross.ac.jp; 4 College of Bioresource Sciences, Nihon University, Tokyo 252-0880, Japan; ono.hiroshi@nihon-u.ac.jp

**Keywords:** dairy farmers, depressive symptoms, work environment factors, gender differences, Japan

## Abstract

Dairy farmers are more likely than nonfarmers to experience high demands and are at risk of mental health problems. However, there is scarce evidence on the current state of psychological health and related factors among dairy farmers, and the knowledge of potential gender differences is limited. This study aimed to examine the prevalence of depressive symptoms assessed using the Center for Epidemiologic Studies Depression Scale (CES-D) and its association with work environment characteristics and to identify gender differences. Data were collected from 273 dairy farmer participants (169 males and 104 females) in Japan. Females were more likely to be depressed, and young and middle-aged women appeared to be at risk of depression. For both genders, a demanding work environment was related to depression. There were some gender differences; for example, worries about the harmful effects of pesticides on health and the balancing of family roles and work roles were related to depression in men, while worries about one’s financial situation and the health status of livestock were associated with depression in women. Females benefited from support through direct interaction, while males benefited from involvement in social activities. These findings will contribute to the development of a gender-specific approach to promote psychological health in the dairy farming community.

## 1. Introduction

In many industrialized countries, agricultural workers have been reported to be decreasing [1]. Japan has shown the same trend; a national report showed that the number of farmers was approximately 3.4 million in 1995, but 2.2 million in 2018 [2]. The industrialization and mechanization of farms has led to a scaled expansion of farmland and a smaller workforce. A great concern exists regarding the shortage in the workforce due to an imbalance caused by the abandonment of farming, the decreasing number of new farmers and a lack of successors to continue to operate farms. For instance, over the past 10 years, in Hokkaido Prefecture, the average of number of agriculture separations was more than 600 per year, which is estimated to be approximately five times higher than the number of new farmers [3,4]. Additionally, government agricultural policy has had a great impact on sustaining farming practice. These data indicate that contemporary farming is being shaped in multi-scalar ways that impact the psychological health of farmers. It is an important global issue to sustain and stably develop agriculture, and the maintenance of farmers’ psychological health could be one of the critical factors to achieve this.

Farmers tend to be more prone than the nonfarming population to exposure to various stressors, including high workload, safety concerns, climate change, government policies and financial concerns [5,6,7]. Farmers are reported to be at risk of mental health problems. Previous studies have shown that farmers have higher psychological morbidity than the nonfarming population [8,9,10]. According to a recent review, several factors, such as pesticide exposure, financial pressures, and climate variability, are risk factors for mental health [5].

Among farmers, dairy farmers often work under unique working conditions. According to a previous study, dairy farmers are more likely to experience high demands, such as heavy workload, time pressure and economic concerns, than non-dairy farmers [7]. Dairy farmers milk cows twice a day without rest, which differs from other types of livestock farming; therefore, dairy farming is characterized as difficult work that must be conducted throughout the year [11]. They also take responsibility for the welfare and health of their livestock every day and animal health seems to be associated with the farmers’ health [12]. Traditional farmers, that is, those who operate their farms based on family, marriage or blood relationships and have smaller farms, may have more difficulty separating their professional and private lives, because their workplaces and homes are combined. They often work from the early morning to late evening, 365 days a year [7]. Longer working hours have also been reported among dairy farmers in Japan: the workload has been estimated to be 2847 hours per year [13], based on a government report [14], compared to an average of 1719 hours for Japanese workers [15]. Nevertheless, the average amount of time that farm helpers were employed was only 22 days per year for a dairy farm household [16]. In addition, the Trans-Pacific Partnership (TPP) agreement adopted in 2018 will possibly lead to a decrease in dairy product prices. In Hokkaido Prefecture, for example, this agreement is estimated to cause milk prices to decrease by a maximum of 16.9 percent and a minimum of 13.2 percent, which is equivalent to 55 billion yen and 42.9 billion yen, respectively, and this is a concern among farmers [17].

A previous study showed that dairy farmers had a higher risk of psychological distress than people with other occupations, including non-dairy farmers [18]. Job demands were reported to be a factor contributing to psychological distress [18], and workload, economic situation and loneliness were related to stress and burnout among dairy farmers [19]. Even though psychological health among dairy farmers is an issue that needs to be investigated, the evidence is still limited, and most research on the topic has been conducted in Western countries and Australia. Building knowledge of diary farmers’ mental issues is essential to develop preventive strategies.

Given that men and women are biologically distinct and can have differing life experiences, gender differences exist in men and women’s responses to life stressors [20]. Evidence has suggested that females have a higher prevalence of depressive symptoms than males [21]. However, knowledge of potential gender differences among dairy farm workers is limited. As described in the literature review, female dairy farmers tend to face difficulties in balancing a variety of tasks and roles at home and work and to have less capacity for physical farm work than men [22]. Additionally, in Japan, women in rural communities are expected to perform more unpaid work, such as housework or childcare, in addition to farm work [23]. It is assumed that an awareness of gender differences will guide the development of a gender-specific approach to promote psychological health among dairy farmers.

The aim of this study was to examine the psychological health and work environment of dairy farmers in Japan. The specific objectives were to determine the prevalence of depressive symptoms, to examine the association of work environment characteristics with depressive symptoms and to identify gender similarities and differences.

## 2. Materials and Methods

### 2.1. Study Design

This study is a cross-sectional study that was conducted from December 2018 to February 2019. We selected two towns located in Hokkaido Prefecture, where dairy farming is a major agricultural industry. Table 1 presents an overview of the study areas [24,25]. For data collection, the research team collaborated with the Japan Agricultural Cooperative (JA), which is an agricultural organization based on the principle of mutual cooperation. This study is part of the project "Evaluation of the dairy farming systems for sustainability using multiple management and health science indicators", which was approved by the Ethics Committee of the Research Faculty of Agriculture, Hokkaido University (approved on October 4, 2018). 

### 2.2. Study Population

The study participants were recruited with cooperation from the JA. The research team members explained the aim of the survey to dairy farmers with written explanation letters and asked them to answer questionnaires if they agreed to participate in the survey. The letter also explained that participants’ return of the questionnaire was considered to indicate consent to participate. The inclusion criteria were dairy farm owners/managers and their families who were involved in dairy farming operations and were aged from 20 to 74. Employed workers were not included in this analysis. Among 289 dairy farm households in both towns, which was estimated based on a previous report [24], 543 questionnaires were distributed, 316 were returned, and 273 of the respondents met the inclusion criteria. 

### 2.3. Measures

Age, gender, marital status, the presence of chronic illness, depressive symptoms, work environment (job demands, job control and work-related worries) and social networks in the community were assessed using questionnaires.

#### 2.3.1. Depressive Symptoms

Depressive symptoms were assessed by the Center for Epidemiologic Studies Depression Scale (CES-D). The CES-D is a 20-item measurement that is commonly used to screen for depression [26]. The reliability and validity of the Japanese version has been confirmed [27]. According to the literature review, the CES-D is one of the instruments that has been used in mental health research with farmers [5]. We used a cut-off point of 15/16; participants who scored 16 or more were defined as having depressive symptoms, as in previous studies.

#### 2.3.2. Job Demands and Job Control

According to the job demand-control model, job demand and low decision latitude are important work factors that increase the risk of psychological health problems [28]. We assessed job demands and job control by using the subscales of the Brief Job Stress Questionnaire (BJSQ). The BJSQ has been shown to be reliable and valid [29] and has commonly been used for regular screening in Japan [30,31]. Job demand was assessed with three items; an example item is “I have an extremely large amount of work to do”. Job control was assessed with three items, including “I can work at my own pace”. These items used a four-point Likert scoring method, with higher scores indicating higher job demands and job control.

#### 2.3.3. Work-Related Worries

Work-related worries were assessed with 15 items. The items were selected based on the previous literature, including the literature on the effect of workload on health, social and relational issues; future, new technology; agricultural policy; animal health; and climate issues [19,32,33,34]. In addition, two items on the following topics were added to reflect current concerns in Japanese agriculture, based on expert opinions: lack of workforce and the availability of a successor to take over the farm. All responses were given on a 4-point scale ranging from “not worried at all” to “very worried”.

#### 2.3.4. Social Networks in the Community

Social networks in the community were assessed with two items. This variable was included because farmers in family dairy farms often have limited social contact [7]. First, work-related support was measured by asking whether farmers had someone with whom they could talk about concerns and worries related to their work. The response items were “yes” or “no”. Social participation was assessed with an 8-item checklist, that inquired whether farmers participated in social activities in the community, such as volunteer activities, residents’ associations or community activities.

### 2.4. Statistical Analyses

Descriptive statistics were generated for all variables, and all analyses were stratified by gender. Responses about work-related worries were dichotomized, because of the ordinal nature of this variable and its applicability to the analysis of data, as “worried”, which included responses of “very worried” and “somewhat worried”, and “not worried”, which included responses of “not too worried” and “not worried at all”. Chi-square tests and t-tests were used for comparisons between men and women. The chi-square test followed by post hoc adjusted residual analysis was used when more than two groups were compared. To examine work environment factors related to depressive symptoms, multiple logistic regression adjusted for age, marital status and chronic health problems was performed by gender, and the results are reported as the odds ratios (ORs) and 95% confidence intervals (95% CIs). All statistical analyses were performed using SPSS version 22.0. The level of significance was set at *p* < 0.05.

## 3. Results

Of the 273 participants, 169 were male, and 104 were female. As shown in Table 2, the mean age was 49.4 years for male participants and 51.0 years for female participants, and there was no significant difference in age between the two groups. Among the participants, 84.4% of males and 95.2% of females were married, and 40.7% of males and 46.5% of females had chronic health problems.

**Figure 1:**
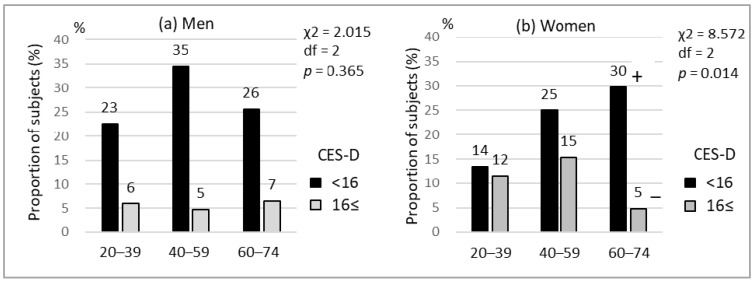
The percentage of subjects with depressive symptoms in each age group: (**a**) Analysis of men; (**b**) Analysis of women. Three age groups (20–39, 40–59, and 60–74) were compared by the chi-square test and subsequently by post hoc adjusted residual analysis. +; adjusted residual >1.96, −; adjusted residual < −1.96.

The prevalence of depressive symptoms differed significantly by gender: 17.3% for males and 31.7% for females. The prevalence of depressive symptoms by age group is shown in Figure 1. For females only, the prevalence of depressive symptoms varied significantly among age groups (*p* = 0.014); the adjusted residual analysis showed that the 60–74 age group was less at risk of depressive symptoms than the 20–39 and 40–59 age groups.

**Figure 2:**
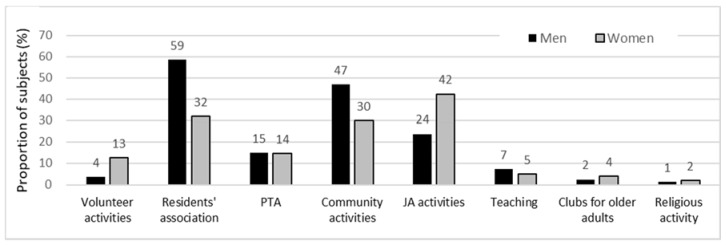
The percentage of the subjects who participated in men’s and women’s social activities. PTA = Parent Teachers Association; JA = Japan Agricultural Cooperative

Table 3 describes work environment characteristics by gender. The job demand scores did not differ between genders (*p* = 0.104), but the job control scores were higher in males than in females (3.4 and 2.8, respectively). Most work-related worries did not differ between genders, but female participants were significantly more worried than male participants about the negative effects of work overload on one’s own health (71.3% in women, 53.9% in men), balancing of family roles and work roles (64.3% in women, 49.4% in men), distance from public facilities (77.5% in women, 63.3% in men) and health status of livestock (66.7% in women, 49.1% in men). Male participants were more engaged in social activities than females. Figure 2 describes the social activities of men and women.

The results of the logistic regression analysis of the effect of work environment factors on depressive symptoms are shown in Table 4. In men, depressive symptoms were associated with higher job demand (OR 2.16; 95% CI 1.17–4.00) and lower job control (OR 0.48; 95% CI 0.25–0.95). Worries about the harmful effects of pesticides on health (OR 2.61; 95% CI 1.06–6.43), the balancing of family roles and work roles (OR 4.04; 95% CI 1.60–10.23) and the lack of workforce (OR 4.55; 95% CI 1.48–14.01) were associated with depressive symptoms. Moreover, social participation was related to a lower risk of depressive symptoms (OR 0.23; 95% CI 0.08–0.66). In women, higher job demand was a risk factor for depressive symptoms (OR 2.39; 95% CI 1.23–4.64). Worries about the negative effects of work overload on one’s own health (OR 3.49; 95% CI 1.03–11.83), a lack of workforce (OR 3.84; 95% CI 1.15–12.78), one’s financial situation (OR 4.99; 95% CI 1.90–13.11), one’s understanding of new information and technology (OR 2.81; 95% CI 1.08–7.28), the future of one’s own farm (OR 2.88; 95% CI 1.13–7.37), and the health status of livestock (OR 4.20; 95% CI 1.36–13.00) were also associated with depressive symptoms. In contrast, having work-related support was related to a lower risk of depressive symptoms (OR 0.11; 95% CI 0.02–0.76).

## 4. Discussion

Physiological problems can not only lead to poor health outcomes in farmers themselves, but also have consequences for the sustainability of agriculture. Relatively few studies have addressed the psychological states of dairy farmers, and we found that females were more depressed than men, that work environment factors were related to depressive symptoms, and that there were slight differences by gender.

In this study, the prevalence rates of depressive symptoms based on CES-D scores of 16 points or more were 17.3% in males and 31.7% in females. While few studies have been conducted on farmers in Japan, the prevalence in males seemed to be relatively lower than those cited in previous evidence, using the CES-D with the same cut off point for workers and middle-aged adults in Japan, which range from 23% to 48% [35,36,37]. Our results also indicated that males had a lower prevalence of depressive symptoms than females, which was consistent with other previous studies [21,38,39]. The lower prevalence of depressive symptoms in male dairy farmers may be due to a protective factor, such as commitment or sense of independence, which may be a distinct characteristic of management positions in farming. Indeed, the previous study in male farmers showed that a sense of independence was important for lowering levels of mental complaints [40]. Our data also showed an association between the prevalence of depressive symptoms and age group among females. Compared to older participants, young and middle-aged participants were at greater risk of depressive symptoms. This finding is consistent with a previous study conducted on Japanese employees in companies [36]. Based on this evidence, attention to this age population is needed to prevent mental health problems.

Before examining the relationship between depressive symptoms and work environmental factors, we compared how work environment factors differed by gender, and we found several differences. The job demand scores did not differ between genders, but the job control scores were higher in males than in females. Most work-related worries did not differ between genders, but female participants were worried significantly more than male participants about the negative effects of work overload on one’s own health, the balancing of family roles and work roles, the distance from public facilities and the health status of livestock. These differences may reflect different capacities for physical work tolerance and different roles between males and females in farming. Women are more likely to be involved in a wide range of different work obligations, that include not only farm work but also household work [22]. Japanese women in particular have traditionally prioritized family work participation [41]. Perhaps this emphasis on family work results in difficulty working at one’s own pace and balancing work and other life roles, as well as more worries about the distance from public facilities, such as the child-rearing environment.

Our results identified that certain work environment factors were associated with depressive symptoms in both genders. According to previous studies, job demands are risk factors for psychological health problems in dairy farmers [18,19]. This study showed an association of job demand with depressive symptoms among men and women, which supported previous findings. Our results also indicated that depressive symptoms were significantly associated with worries about a lack of workforce in both genders. This finding might be explained by the fact that higher job demand often results in emerging concerns about labor force shortages. We hypothesized that job control was a risk factor for depressive symptoms, but our results did not statistically support this hypothesis for female participants.

This study showed some gender differences in the association between work environment characteristics and depressive symptoms. One of the main differences was in the association of work environment characteristics with economic concerns. In women, participants who worried about their financial situations had almost a five times greater risk of depressive symptoms than those who did not. The association of economic concerns with stress or mental health status among farmers in general, as well as in dairy farmers in particular has been identified in previous literature [19,42]. Our study showed that worries about financial situation were related to depressive symptoms only for women. According to the economic literature, there are gender differences in risk-taking behavior, and women appear to be more financially risk averse than men [43]. This gendered tendency was one of the possible explanations for our results, as women appeared to be more vulnerable to financial concerns than men. Otherwise, there might be a psychological factor that buffers mental health impacts among men. This topic should be a focus in future studies. A previous study reported that financial hardship was related to depression among women rice farmers, which is consistent with our results [44].

Female farmers who worried about the health status of livestock were 4.2 times as likely to be depressed than those who did not. This might be a unique result in the dairy farming context. A previous study reported that a high disease incidence rate of cows was associated with more exposure to negative psychosocial work environment factors, and this association was found among employed workers but farm owners/managers [12]. The authors discussed two potential reasons for this association: the physically demanding aspect of employed workers’ work, as well as the mentally demanding aspect of their work, due to empathy or feelings related to the cows when cows had disease. Most likely, the same tendency was observed in this study; women appeared to be more depressed when they experienced physical and psychological demands due to the health status of cows.

Our results indicated that worries about balancing family roles and work roles seemed to be related to depressive symptoms among men, as men who had such worries had a 4 times greater risk of depressive symptoms than those who did not. On the other hand, in women, participants who worried about balancing family roles and work roles were 2.7 times more likely to be depressed, which was not statistically significant. In accordance with previous literature that reported that female dairy farmers tend to face difficulty balancing a variety of tasks and roles at home as well as at work [22], our results showed that women were more likely to be worried about balancing family roles and work roles than men (49.4% and 64.3%, respectively). However, interestingly, it appears that difficulty maintaining role balance had a more negative impact on mental health in men.

The results showed that worries about the harmful effects of pesticides on health were related to depressive symptoms in men; men who had such worries were 2.6 times as likely to be depressed than in those who did not, while there was no association among women. The association between pesticide exposure and depression has been a major concern in previous research [45]. It is understandable that farmers with concerns about the harmful effects of pesticides tended to be prone to depressive symptoms, but we are not sure why only men showed this association. It can be assumed that the extent of exposure might differ between genders.

Work-related support was associated with depressive symptoms for female but not male participants, but social participation was related to depressive symptoms only for male participants. These findings indicated that social relations can be a protective factor against depressive symptoms, but there might be different effects on depressive symptoms for men and women. The literature has revealed that social ties and support have a positive effect on health in general [46]. One study in a sample of an older population showed that women were more likely to benefit from social participation than men [47]. However, social ties can also have negative aspects on mental health, such as through a sense of obligation or demanding social ties in the community, especially for women [46,48]. This might be a possible explanation why social participation was not related to depressive symptoms among women, while women benefited from support from someone with whom they could share concerns and worries through more personal interaction. However, among male participants, being involved in social activities had a positive effect on mental health. This finding is similar to that of a study showing that community integration was negatively associated with depression only for males among rice farmers [44].

There were several limitations of the study. Although the instrument used for this study has been confirmed to be valid in Japan and has been widely used worldwide, caution should be taken in the interpretation of the prevalence of depressive symptoms for the following reasons. First, the results were based on a self-reported questionnaire and were not clinically evaluated. Second, farmers who were depressed may not have been as likely to respond to this survey as farmers who were not depressed. In addition, this study was a cross-sectional study, so we did not identify causal relationships. Finally, the sample was not representative of all dairy farmers in Japan.

Despite the limitations, to our knowledge, this is the first study to focus on depressive symptoms and gender differences in Japan. Important observations were presented that will be helpful for planning appropriate gender-specific strategies, such as awareness programs or intervention in the community to focus on mental health issues. Despite the fact that the female agricultural population accounted for 43 % of the agricultural workforce in 2015 [24], reports have shown that the number of women in in leadership or management positions in agricultural organizations is low; some reports have indicated that this number is about 10 percent or less in these years [49]. One important step may be to equally involve women and men in the decision-making process [23]. This would allow the voices and opinions of both genders to be heard in their community and allow the development of strategies tailored to gender.

## 5. Conclusions

This study focused on the prevalence of depressive symptoms among dairy farmers and examined the association between work environment characteristics and depressive symptoms by gender in the Japanese context. Females were more likely to be depressed, and young and middle-aged women appeared to be at risk of depressive symptoms. Work characteristics related to a high amount of job demand were associated with depressive symptoms in both genders. Among women, participants who worried about their financial situations and who worried about the health status of livestock had an almost 4–5 times greater risk of depressive symptoms than those who did not. While women were more likely to worry about balancing family roles and work roles than men, men who worried about balancing family roles and work were more likely to be at risk of depressive symptoms. The protective factors also differed by gender; females appeared to benefit from support through direct interaction, while involvement in social activities had a positive effect on mental health for men. This study presented some important observations that will be helpful for planning appropriate gender-specific strategies to identify dairy farmers who are at risk for depressive symptoms. The maintenance of psychological health is important for farmers’ health outcomes as well as the sustainability of agriculture in the future. This study will contribute to the knowledge of specific approaches to promote psychological health in the dairy farming community.

## Figures and Tables

**Table 1:** Description of the study areas.

Variables	Japan	Hokkaido Prefecture	Town A	Town B
Family farming (%)	97	90	-	-
Average number of cows per dairy farm household	-	122.9	187.9	118.9
Dairy cow output per gross agricultural output (%)	10	39	63	94

Data were calculated from the reference of Ministry of Agriculture, Forestry and Fisheries [24,25].

**Table 2:** Patient characteristics by gender.

Variables	Men	Women	*p* Value
	*n* = 169	*n* = 104	
Age (years)	49.9 ± 14.0	51.0 ± 13.2	0.517
20–39	48 (28.4)	26 (25.0)	0.824
40–59	66 (39.1)	42 (40.4)	
60–74	55 (32.5)	36 (34.6)	
Married	141 (84.4)	99 (95.2)	0.007 **
Has chronic health problems	68 (40.7)	47 (46.5)	0.351
CES-D (total score)	11.5 ± 7.4	14.3 ± 9.1	0.010 *
16≤	29 (17.3)	33 (31.7)	0.006 **

Results are presented as *n* (%) or mean ± SD. *p*-values were calculated using chi-square tests for categorical variables and *t*-test for continuous variables. * *p* value < 0.05; ** *p* value < 0.01.

**Table 3:** Description of work environment characteristics by gender.

Variables	Men *n* = 169	Women *n* = 104	*p* Value
Job demand	2.7 ± 0.8	2.5 ± 0.8	0.104
Job control	3.4 ± 0.6	2.8 ± 0.7	<0.001 **
Work-related worries, *n* (%)			
Farm related accidents and injuries	124 (75.6)	78 (77.2)	0.764
Harmful effects of pesticide on health	42 (25.6)	37 (36.6)	0.057
Negative effects of work overload on own health	89 (53.9)	72 (71.3)	0.005 **
Negative effects of work overload on family’s’ health	115 (69.7)	82 (80.4)	0.054
Balancing family role and work role	81 (49.4)	63 (64.3)	0.019 *
Maintain relationships with farmers in the neighborhood	45 (27.3)	28 (27.5)	0.975
Lack of workforce	102 (61.4)	70 (69.3)	0.193
Financial situation	73 (44.5)	43 (43.0)	0.810
Understanding new information and technology	73 (44.2)	54 (54.0)	0.123
Successor to take over the farm	54 (32.9)	40 (39.2)	0.297
Future of own farm	82 (50.0)	54 (52.9)	0.641
Agricultural policy in Japan	137 (83.0)	81 (81.8)	0.802
Distance from public facilities such as hospitals/schools	105 (63.3)	79 (77.5)	0.015 *
Health status of livestock	81 (49.1)	66 (66.7)	0.005 **
Disaster	140 (84.3)	92 (90.2)	0.172
Work-related support	151 (91.5)	92 (92.0)	0.890
Social participation	148 (89.2)	81 (77.9)	0.012 *

Results are presented as *n* (%) or mean ± SD. *p*-values were calculated using chi-square tests for categorical variables and *t*-test for continuous variables. * *p* value < 0.05; ** *p* value < 0.01.

**Table 4:** Logistic regression analysis of work environment factors relating to depressive symptoms.

Variables	Men *n* = 169			Women *n* = 104	
	OR (95%CI)	*p* Value		OR 95%CI	*p* Value
Job demand	2.16 (1.17–4.00)	0.014 *		2.39 (1.23–4.64)	0.010 *
Job control	0.48 (0.25–0.95)	0.034 *		0.57 (0.29–1.13)	0.107
Work-related worries					
Farm related accidents and injuries	1.29 (0.48–3.48)	0.610		1.59 (0.51–5.00)	0.429
Harmful effects of pesticide on health	2.61 (1.06–6.43)	0.037 *		1.99 (0.80–4.97)	0.138
Negative effects of work overload on own health	2.16 (0.91–5.14)	0.083		3.49 (1.03–11.83)	0.045 *
Negative effects of work overload on family’s’ health	1.77 (0.66–4.72)	0.258		3.67 (0.76–17.66)	0.104
Balancing family role and work role	4.04 (1.60–10.23)	0.003 **		2.69 (0.95–7.66)	0.064
Maintain relationships with farmers in the neighborhood	2.17 (0.89–5.25)	0.087		2.04 (0.76–5.45)	0.156
Lack of workforce	4.55 (1.48–14.01)	0.008 **		3.84 (1.15–12.78)	0.029 *
Financial situation	1.70 (0.73–3.99)	0.222		4.99 (1.90–13.11)	0.001 **
Understanding new information and technology	1.96 (0.85–4.50)	0.113		2.81 (1.08–7.28)	0.034 *
Successor to take over the farm	1.21 (0.51–2.84)	0.669		1.75 (0.71–4.28)	0.222
Future of own farm	2.24 (0.95–5.29)	0.065		2.88 (1.13–7.37)	0.027 *
Agricultural policy in Japan	2.18 (0.60–7.95)	0.240		1.43 (0.43–4.77)	0.565
Distance from public facilities such as hospitals/schools	0.95 (0.41–2.20)	0.991		1.56 (0.50–4.90)	0.447
Health status of livestock	2.07 (0.86–4.95)	0.103		4.20 (1.36–13.00)	0.013 *
Disaster	0.96 (0.31–2.94)	0.938		0.74 (0.15–3.55)	0.705
Work-related support	0.63 (0.15–2.76)	0.542		0.11 (0.02–0.76)	0.025 *
Social participation	0.23 (0.08–0.66)	0.007 **		0.46 (0.16–1.34)	0.155

OR = odds ratio; CI = confidence interval. Each model was adjusted for age, marital status and chronic health problem. * *p* value < 0.05; ** *p* value < 0.01.
